# Titanium- and Strontium-Infused Calcium Silicates Toward Restorative and Regenerative Purposes

**DOI:** 10.7759/cureus.60863

**Published:** 2024-05-22

**Authors:** Tanu Patel, Chitra S, Reenu Joshy, Pasiyappazham Ramasamy, Dhanraj Ganapathy

**Affiliations:** 1 Prosthodontics, Saveetha Dental College and Hospitals, Saveetha Institute of Medical and Technical Sciences, Saveetha University, Chennai, IND

**Keywords:** strontium infused cements, titanium, regenerative cements, bioactive material, calcium silicate cement

## Abstract

Background: Dental materials with dentine regenerative properties are preferred over conventional materials. Calcium silicate cements, such as Biodentine, are bioactive and offer excellent sealing ability, making them ideal for various dental treatments.

Objectives: This study aimed to fabricate bioactive calcium silicates infused with titanium (Ti) and strontium (Sr) to optimize their neo-angiogenic, antimicrobial, and regenerative properties while maintaining mechanical stability.

Methodology: Ti- and Sr-infused calcium silicate cements were synthesized, and their mineral phases were characterized using X-ray diffraction. Morphological and elemental analyses were performed using field emission scanning electron microscopy (FESEM) and energy dispersive X-ray spectroscopy (EDS). Raman spectroscopy was used to confirm the formation of bioactive material. A hemocompatibility assessment was conducted to evaluate blood compatibility.

Results: The presence of Ca_2_, SiO_4_, and SrTiO_3_ mineral phases indicated the successful infusion of Ti and Sr into the calcium silicate cement. FESEM and EDS revealed interconnected small spheres and rods in the silicate network with the relevant elemental compositions. Raman spectra verified that Si-O-Si and Ti-O-Ti vibrations exist, validating the formation of a bioactive material. The hemocompatibility assessment demonstrated optimal blood compatibility.

Conclusions: This study successfully fabricated an improved calcium silicate-based material with enhanced regenerative properties and excellent biocompatibility. This newly formed substrate holds promise for providing superior restorative solutions and aiding in conservative treatment modalities during dental procedures.

## Introduction

Calcium silicate-based cements are spontaneously setting hydraulic cements that are used for a wide range of restorative and regenerative purposes in dentistry. It is a unique dental material because of its hydraulic properties, which promote spontaneous setting when exposed to water and can continuously release calcium for a considerable amount of time after setting [1]. There are several properties of calcium silicate cement, making it suitable as a restorative material for clinical applications such as its ability for initial expansion by a volume of 0.2% to 6%, leading to a tight seal and thus decreasing micro-leakage [2]. In the presence of water, the setting time was 40 to 120 minutes, with an initial setting time of approximately 40 to 50 minutes and a final setting time of 120 to 170 minutes. The presence of body fluid containing protein, along with the pH and pressure used to crush the cement during repair, have an impact on the setting time. Owing to their capacity to release calcium hydroxide to produce an alkaline environment that damages DNA, cytoplasmic membranes, and proteins, they also have antibacterial activity. Additionally, it has the extraordinary capacity to initiate apatite crystals and functions favorably as a promoter of cell differentiation and a stimulant of tissue healing, bone formation, and cementum formation [2]. Despite its numerous advantageous properties for restorative applications, this substance has drawbacks such as low radiopacity, requiring the addition of radiopacifiers which can reduce its biocompatibility, and its high solubility, ranging from 12% to 38%, which increases the risk of micro-leakage.

Calcium silicates are widely used in reconstructive and restorative dentistry because of their stimulatory effect on stem cells from the dental pulp and stromal cells, which encourages the formation of freshly generated reparative dentin [3]. It may be applied to manage tooth hypersensitivity, apicogenesis, apexification, pulp revascularization, treatment of open apex roots, root tip sealing substances, endodontic treatment in primary teeth, and dentinal remineralization [4,5]. In earlier studies, titanium (Ti)-modified bioactive glasses and Ti oxide-doped hydroxyapatite showed significant enhancements in osteoblast proliferation, differentiation, and mineralization [6]. Since the 20th century, research into the health benefits of strontium (Sr) has been conducted, particularly focusing on its potential impact on bone health. Sr, situated in the same group as calcium (Ca) in the periodic table, has garnered attention for its ability to influence the formation of new bone tissue. This interest stems from its capability to substitute calcium within bone crystals, thereby affecting bone metabolism. Moreover, Sr exhibits an inductive effect on osteoblasts, the cells responsible for bone formation, while simultaneously exerting a suppressive effect on osteoclasts, which are involved in bone resorption. These dual actions contribute to Sr's potential to promote bone regeneration and density.

Beyond its role in bone health, Sr possesses notable antibacterial properties and has been associated with tissue regeneration. In dental applications, Sr has demonstrated various beneficial effects, including the upregulation of odontoblasts (cells responsible for dentin formation), enhancement of radiopacity (which aids in dental imaging), management of dentinal hypersensitivity, and inhibition of caries formation. Due to these advantageous properties, Sr has become a desirable ingredient in dental materials. The objective of the present study was to enhance the properties of calcium silicate-based dental materials by incorporating Ti and Sr. By infusing these materials with Ti and Sr, the aim was to optimize their ability to stimulate the formation of new blood vessels (neo-angiogenesis), exhibit antimicrobial activity, and promote tissue regeneration. Importantly, the study sought to achieve these enhancements while ensuring that the mechanical stability of the restorative material was maintained.

## Materials and methods

Materials

All the reagents and chemicals employed in this study were of analytical grade and were used without any further purification. We bought nitric acid or tetraethyl orthosilicate (TEOS Alfa Aesar) from Spectrum Reagents and Chemicals Pvt. Ltd. in Kerala, India. Silver nitrate and zinc nitrate were acquired from Sisco Research Laboratories Pvt. Ltd (SRL), and calcium nitrate was purchased from Merck.

Synthesis protocol

Calcium silicate was created using the sol-gel process with the following compositions: SiO (0.3 M), CaO (0.3 M), ZnO (0.1 M), and AgO (0.1 M). Tetraethyl orthosilicate (1.37 mL) was used to prepare the calcium silicate bioactive materials. This was mixed in ethanol (5 mL), double-distilled water (DD H_2_O) combination (10 mL), and nitric acid (3 mL) was administered as a catalyst to accelerate the gelation process. Following the creation of the whole gel, 10 mL of DD H_2_O was used to dissolve calcium nitrate (0.708 g), which was then added to the basic silicate matrix. Similarly, within an hour of each other's dissolution in DD H_2_O, zinc nitrate (0.298 g) and silver nitrate (0.17 g) were added to the base material. The fabricated sol was first heated to 600 °C to stabilize the ingredients and then allowed to dry in a hot air furnace at 100 °C for 24 h.

Characterization techniques

X-ray diffraction (XRD) patterns were utilized to evaluate the crystalline phases with the wavelength of Cu K (Bruker D8 advance) and characterize the properties of the synthesized calcium silicate. Raman spectroscopy (WITEC ALPHA300 RA - Confocal Raman Microscope with AFM) was used to examine the functional group characteristics. Oxford Instruments and JEOL (JSM-IT 800) conducted morphological and elemental analyses, respectively.

Blood compatibility assessment

To assess the compatibility of bioactive materials with blood cells, erythrocyte compatibility was assessed. The anticoagulant ethylenediaminetetraacetic acid (EDTA) was used to keep the blood from coagulating after it was drawn from the volunteer. Once red blood cells (RBCs) were recovered, the blood components were removed by centrifuging the RBCs at 4 °C for 10 minutes. They then washed them three times in phosphate-buffered saline (PBS) with a pH of 7.4. The erythrocyte rupture rate (RBCs lysis behavior) was calculated using a hemocompatibility assay and compared with the negative and positive controls. Three replicates of each concentration of bioactive material were examined. One hour was spent incubating each test sample at 37 ℃. After centrifuging erythrocytes that had been treated with bioactive material, the rate of erythrocyte rupture was determined at a wavelength of 540 nm (Equation 1). Approval from the committee on ethics (IHEC/SDC/FACULTY/23/PROSTHO/208) was obtained to ensure blood compatibility.

Hemolysis % = Sample absorbance - Negative control/Positive control - Negative control x 100..........(Equation 1)

## Results

X-ray diffraction

**Figure 1:**
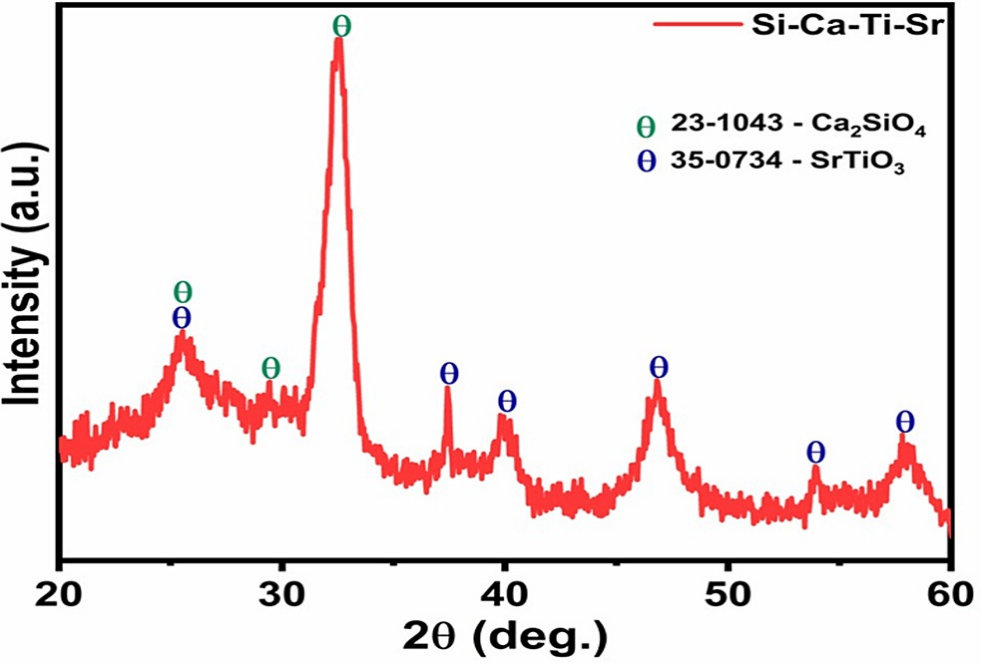
X-ray diffraction pattern indicating titanium- and strontium-infused calcium silicate.

X-ray diffraction is used to analyze the crystal structure of the bioactive material obtained by Ti and Sr infusion. Figure 1 shows the diffracted peaks in the X-ray diffraction pattern, which confirms the formation of Ca_2_SiO_4_ and SrTiO_3_ mineral phases on the host material. Sr, Ti oxide, and calcium silicate mineral phases are indicated with their respective colors with JSPDS card numbers in the graph. The graph shows multiple minor and major peaks, each representing the formation of a crystal phase, with the highest intensity peak associated with Ca_2_SiO and multiple peaks representing the formation of SrTiO_3_ at the sintering temperature, indicating successful infusion and the existence of both crystal structures together.

Raman spectra

**Figure 2:**
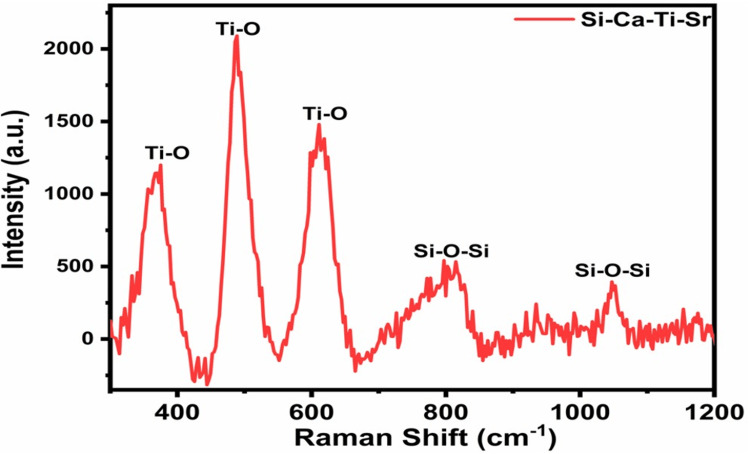
Raman spectra of titanium- and strontium-infused calcium silicate.

It was used to determine the vibrational modes of the oxides with the relevant metal ions. Figure 2 shows the infusion of Ti and Sr into the calcium silicate. Raman spectra explained the Ti-O-Ti stretching as well as Si vibrational modes, thus confirming the formation of bioactive materials. These results further supported the diffraction patterns obtained in previous studies.

Morphological and elemental analysis

**Figure 3:**
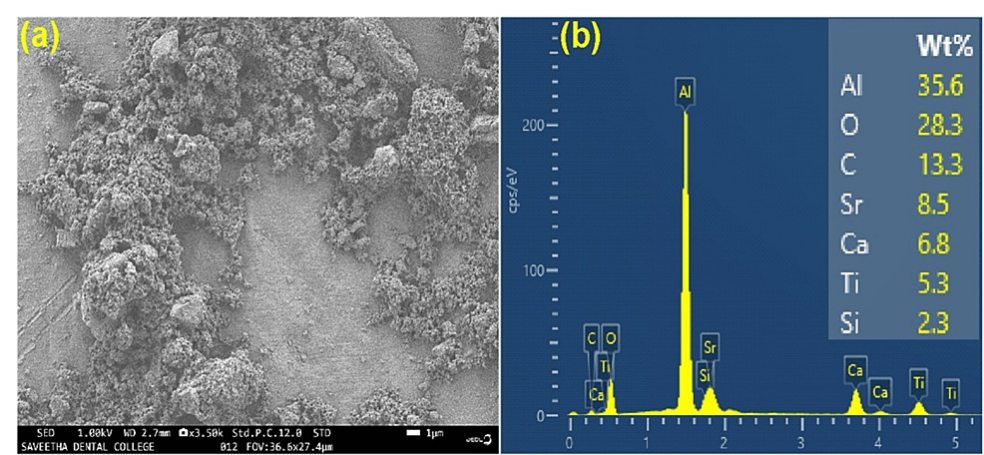
Morphological and elemental analysis of strontium- and titanium-infused calcium silicate through (a) field emission scanning electron microscopy (FESEM) and (b) energy-dispersive X-ray spectroscopy (EDX).

FESEM and EDS were used to analyze the morphological as well as elemental properties of the synthesized material. Figure 3 shows the morphological analysis of Sr- and Ti-infused calcium silicates. Interconnected small spheres and rods were visible on the silicate network, along with relevant elemental compositions such as O, C, Sr, Ca, Ti, and Si via Raman spectra. An intense aluminum peak was observed because of the base component of the aluminum foil.

Hemocompatibility assessment

**Figure 4:**
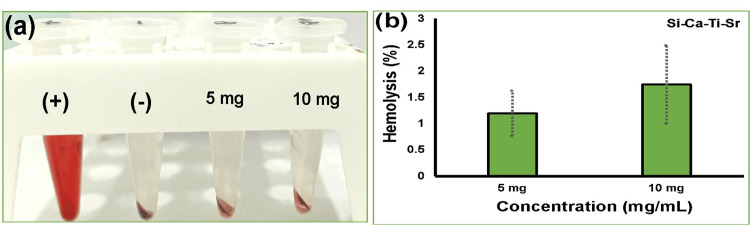
Hemocompatibility assessment of strontium- and titanium-infused calcium silicate: (a) centrifuge tubes and (b) histogram showing lysis percentage, representing the photographic image of hemolysis (with concentrations of 5 and 10 mg, along with positive and negative control).

Figure 4 shows the blood compatibility of the synthesized Ti- and Sr-infused calcium silicates. It also represents the Si-Ca-Ti-Sr bioactive materials compatibility with erythrocytes explicates below 2% lysis at a concentration of 10 mg/mL, which is acceptable as the American Society for Testing and Materials (ASTM) standard. Since the maximum lysis of the material interaction with blood cells was up to 5%, according to the standards, it is considered a compatible material for biomedical applications.

## Discussion

Calcium silicates are promising dental materials for various potential therapeutic applications. The development of biodentine expanded the indications of calcium silicate cement such as endodontic procedures involving dentin and cementum repair, pulp regeneration, management of external root resorption, base beneath a resin composite, and also as intermediate restoration [7]. Recently, many adhesive dental restorative materials were believed to interact passively with the enamel/dentin they were placed on, depending on the infiltration of the material [8]. Materials that express a particular biological reaction at the interface between the material and tissue, resulting in the establishment of a bond, are considered bioactive materials. Therefore, despite the presence of these active interactions for decades, its understanding is very recent due to insufficient data available the availability of apt technology previously available to the researchers, and a dearth of research being conducted in this background. Therefore, the evidence available to us is still insufficient to indicate the probable chemical interaction between both resin and water-based cement materials such as calcium silicate cement.

Calcium silicate cements are considered potent bioactive materials for regenerating bone tissue that can be enhanced by the infusion of Sr, which also gives antibacterial characteristics to the material. However, calcium silicate ceramics also have the major drawback of high dissolution, which leads to poor mechanical stability and hence alkalinization in the surrounding environment, limiting their biological application. Thus, Ti can be incorporated to improve the chemical stability of the host material. According to the calcium silicate crystal structure, Quadrivalent Ti4+ can connect to the network to form a more stable tetrahedral network with Si, O, and Ca ions. Previous research has demonstrated that it might increase the rate at which an implant is integrated with osseous tissue, and changing the interfacial chemistry of biomaterials is recognized to be crucial for bone formation [9,10]. Thus, in this study, we aimed to infuse Ti and Sr with calcium silicate to enhance the restorative, regenerative, and antimicrobial activity, which further increases the scope of materials in this field and studies the interaction of the improved material with the dental tissue. Thus, the obtained material eliminates the limitations of conventional calcium silicate and improves the overall biochemical properties of this cement for application in treatment procedures. There is future scope for further in-vivo studies with this material to assess its advantages over conventional calcium silicate so that this material is accepted for its clinical use in routine procedures.

**Figure 5:**
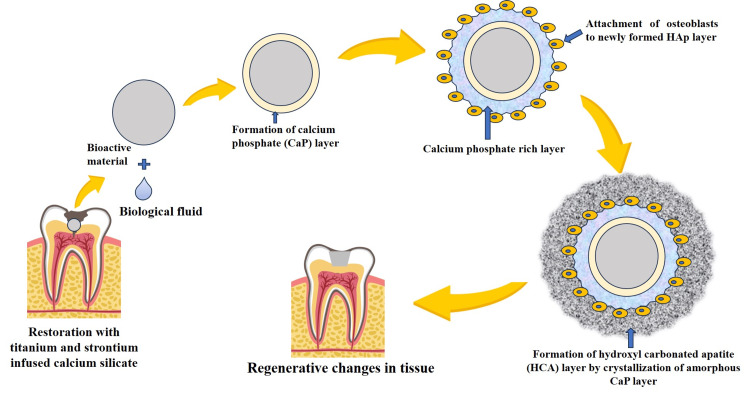
Schematic representation of bioactive calcium silicate materials for the regeneration of alveolar bone. Image credit: All authors.

Figure 5 graphically demonstrates that the obtained material shows properties similar to those of conventional calcium silicate cement, which when in contact with body fluid, shows the formation of calcium phosphate, which further attracts osteoblasts to attach to the newly formed hydroxyapatite crystals formed by the interaction of bioactive material with body fluid. This reaction leads to the formation of a hydroxyl-carbonated apatite layer via the crystallization of amorphous calcium phosphate, thus forming a bone-like structure [11]. This bone regeneration was further enhanced by the infusion of Sr into calcium silicate, as Sr has been shown to stimulate proliferation.

Differentiation and decreased apoptosis of osteoblasts accelerate bone formation and reduce bone resorption as it causes apoptosis of osteoclasts, which will help reduce the healing period and hasten the process of bone formation. Thus, we can expect this material to be used as an advanced material for implant surface coating to reduce the time needed for osseointegration and early loading of the implants [12-15]. Along with this, it is expected that the bioactive material obtained will exhibit improved biocompatibility and mechanical stability, aided by the incorporation of Ti. Additionally, the material is anticipated to possess enhanced regenerative, angiogenic, and antimicrobial properties due to the addition of Sr, which is beneficial for maintaining a sterile environment. These attributes make it suitable for various prosthetic rehabilitations, such as implants, as well as endodontic regenerative procedures like apexogenesis and apexification. This wider range of applications extends to basic restorative and regenerative procedures, including root-end filling, direct and indirect pulp capping, root canal sealing, treatment of open-ended root apices in immature teeth, pulpectomy, pulpotomy, management of dentinal hypersensitivity and remineralization, and bone regeneration [16-20].

Limitations and future perspectives

Calcium silicates are one of the most widely applicable dental materials with a wide range of applications in various disciplines of dentistry, with the most commonly used areas being restorative and regenerative dentistry. This study intends to reflect upon the properties of conventional calcium silicates and why and how to enhance the properties such that the shortcomings of the material can be balanced by incorporating materials such as Ti and Sr. The formation of the desired material has been proven by various tests, and its biocompatibility has been assessed by a hemocompatibility assay. The formulation of this material is expected to enhance its restorative, regenerative, and antimicrobial properties. It also has improved chemical stability and sealing ability. This bioactive material is expected to be used in the field of regeneration, where it can be coated over implants, reducing the time of osseointegration, apexogenesis, and apexification, and serving as root-end filling material for the regeneration of the open apex. It can also aid in direct and indirect pulp capping, facilitating the formation of reparative dentin, as well as address root resorption, perforation repair, etc.

## Conclusions

This study effectively developed an advanced calcium silicate-based material with enhanced regenerative characteristics and exceptional biocompatibility. This newly created material has the potential to deliver superior restorative options and support conservative treatment approaches in dental procedures. Unlike traditional options such as mineral trioxide aggregate (MTA), this novel formulation offers heightened regenerative capabilities, promoting tissue repair in damaged dental structures like dentin, pulp, and periapical areas. Its exceptional biocompatibility ensures minimal tissue irritation and inflammation, particularly crucial in procedures involving direct contact with vital tissues. By facilitating more conservative treatment approaches and expanding restorative options, this material holds promise for improving outcomes and patient satisfaction in dental care, marking a significant leap forward in the field.

## References

[1] Sun Q, Gustin JW, Tian FC, Sidow SJ, Bergeron BE, Ma JZ, Tay FR (2021). Effects of pre-mixed hydraulic calcium silicate putties on osteogenic differentiation of human dental pulp stem cells in vitro. J Dent.

[2] Hiraishi N, Yiu CK, King NM, Tay FR (2009). Antibacterial effect of experimental chlorhexidine-releasing polymethyl methacrylate-based root canal sealers. J Endod.

[3] Primus C, Gutmann JL, Tay FR, Fuks AB (2022). Calcium silicate and calcium aluminate cements for dentistry reviewed. J Am Ceram Soc.

[4] Seo DG, Lee D, Kim YM, Song D, Kim SY (2019). Biocompatibility and mineralization activity of three calcium silicate-based root canal sealers compared to conventional resin-based sealer in human dental pulp stem cells. Materials (Basel).

[5] Singh M, Shivalingam C, Blessy S, Sekaran S, Sasanka K, Ganapathy D (2023). Zinc and silver-infused calcium silicate cement: unveiling physicochemical properties and in vitro biocompatibility. Cureus.

[6] Andrei M, Vacaru RP, Coricovac A, Ilinca R, Didilescu AC, Demetrescu I (2021). The effect of calcium-silicate cements on reparative dentinogenesis following direct pulp capping on animal models. Molecules.

[7] Tohma A, Ohkura N, Yoshiba K (2020). Glucose transporter 2 and 4 are involved in glucose supply during pulpal wound healing after pulpotomy with mineral trioxide aggregate in rat molars. J Endod.

[8] Makarov R, Sintsov M, Valeeva G, Starikov P, Negrov D, Khazipov R (2021). Bone conducted responses in the neonatal rat auditory cortex. Sci Rep.

[9] Balbinot G de S, Leitune VCB, Nunes JS, Visioli F, Collares FM (2020). Synthesis of sol-gel derived calcium silicate particles and development of a bioactive endodontic cement. Dent Mater.

[10] Talabani RM, Garib BT, Masaeli R (2020). Bioactivity and physicochemical properties of three calcium silicate-based cements: an in vitro study. Biomed Res Int.

[11] Shu Y, Ma M, Pan X, Shafiq M, Yu H, Chen H (2023). Cobalt protoporphyrin-induced nano-self-assembly for CT imaging, magnetic-guidance, and antioxidative protection of stem cells in pulmonary fibrosis treatment. Bioact Mater.

[12] Bossù M, Mancini P, Bruni E (2021). Biocompatibility and antibiofilm properties of calcium silicate-based cements: an in vitro evaluation and report of two clinical cases. Biology (Basel).

[13] Cheng D, Ding R, Jin X (2023). Strontium ion-functionalized nano-hydroxyapatite/chitosan composite microspheres promote osteogenesis and angiogenesis for bone regeneration. ACS Appl Mater Interfaces.

[14] Liu H, Gu R, Li W (2023). Engineering 3D-printed strontium-titanium scaffold-Integrated highly bioactive serum exosomes for critical bone defects by osteogenesis and angiogenesis. ACS Appl Mater Interfaces.

[15] Yuan X, Wu T, Lu T, Ye J (2023). Effects of zinc and strontium doping on in vitro osteogenesis and angiogenesis of calcium silicate/calcium phosphate cement. ACS Biomater Sci Eng.

[16] Liu X, Huang H, Zhang J, Sun T, Zhang W, Li Z (2023). Recent advance of strontium functionalized in biomaterials for bone regeneration. Bioengineering (Basel).

[17] Alfahlawy A, Selim MA, Hassan HY (2023). Biocompatibility of three different root canal sealers, experimental study. BMC Oral Health.

[18] Gaber MS (2023). Pulp capping materials and effect of biomaterials on angiogenesis. Biomat J.

[19] Talebi S, Nourbakhsh N, Talebi A (2024). Hard tissue formation in pulpotomized primary teeth in dogs with nanomaterials MCM-48 and MCM-48/hydroxyapatite: an in vivo animal study. BMC Oral Health.

[20] Chen W, Zhang C, Peng S, Lin Y, Ye Z (2023). Hydrogels in dental medicine. Adv Ther.

